# User-Centered Design of A Novel Risk Prediction Behavior Change Tool Augmented With an Artificial Intelligence Engine (MyDiabetesIQ): A Sociotechnical Systems Approach

**DOI:** 10.2196/29973

**Published:** 2022-02-08

**Authors:** Cathy Shields, Scott G Cunningham, Deborah J Wake, Evridiki Fioratou, Doogie Brodie, Sam Philip, Nicholas T Conway

**Affiliations:** 1 Division of Population Health and Genomics School of Medicine University of Dundee Dundee United Kingdom; 2 Usher Institute University of Edinburgh Edinburgh United Kingdom; 3 Ninewells Hospital and Medical School University of Dundee Dundee United Kingdom; 4 MyWay Digital Health Dundee United Kingdom; 5 Grampian Diabetes Research Unit Aberdeen Royal Infirmary Aberdeen United Kingdom; 6 NHS Tayside Dundee United Kingdom

**Keywords:** diabetes mellitus, digital health intervention, eHealth, artificial intelligence, user-centred design, human factors, think aloud

## Abstract

**Background:**

Diabetes and its complications account for 10% of annual health care spending in the United Kingdom. Digital health care interventions (DHIs) can provide scalable care, fostering diabetes self-management and reducing the risk of complications. Tailorability (providing personalized interventions) and usability are key to DHI engagement/effectiveness. User-centered design of DHIs (aligning features to end users’ needs) can generate more usable interventions, avoiding unintended consequences and improving user engagement.

**Objective:**

MyDiabetesIQ (MDIQ) is an artificial intelligence engine intended to predict users’ diabetes complications risk. It will underpin a user interface in which users will alter lifestyle parameters to see the impact on their future risks. MDIQ will link to an existing DHI, My Diabetes My Way (MDMW). We describe the user-centered design of the user interface of MDIQ as informed by human factors engineering.

**Methods:**

Current users of MDMW were invited to take part in focus groups to gather their insights about users being shown their likelihood of developing diabetes-related complications and any risks they perceived from using MDIQ. Findings from focus groups informed the development of a prototype MDIQ interface, which was then user-tested through the “think aloud” method, in which users speak aloud about their thoughts/impressions while performing prescribed tasks. Focus group and think aloud transcripts were analyzed thematically, using a combination of inductive and deductive analysis. For think aloud data, a sociotechnical model was used as a framework for thematic analysis.

**Results:**

Focus group participants (n=8) felt that some users could become anxious when shown their future complications risks. They highlighted the importance of easy navigation, jargon avoidance, and the use of positive/encouraging language. User testing of the prototype site through think aloud sessions (n=7) highlighted several usability issues. Issues included confusing visual cues and confusion over whether user-updated information fed back to health care teams. Some issues could be compounded for users with limited digital skills. Results from the focus groups and think aloud workshops were used in the development of a live MDIQ platform.

**Conclusions:**

Acting on the input of end users at each iterative stage of a digital tool’s development can help to prioritize users throughout the design process, ensuring the alignment of DHI features with user needs. The use of the sociotechnical framework encouraged the consideration of interactions between different sociotechnical dimensions in finding solutions to issues, for example, avoiding the exclusion of users with limited digital skills. Based on user feedback, the tool could scaffold good goal setting, allowing users to balance their palatable future complications risk against acceptable lifestyle changes. Optimal control of diabetes relies heavily on self-management. Tools such as MDMW/ MDIQ can offer personalized support for self-management alongside access to users’ electronic health records, potentially helping to delay or reduce long-term complications, thereby providing significant reductions in health care costs.

## Introduction

Diabetes is a chronic disease affecting an estimated 463 million adults worldwide [1], with global spending related to diabetes and its complications exceeding US $800 billion annually [2]. Good control of diabetes decreases the risk of associated chronic complications [3]. Digital health interventions (DHIs), delivered through interactive websites and mobile apps, have the potential to harness the omnipresence and growing computational power of electronic devices for the self-management of chronic diseases [4-6]. Usability and “tailorability” are known to improve user acceptability and engagement with DHIs [7,8] and, therefore, potentially, their effectiveness. A relatively small number of studies have so far been published on DHI usability [9]*,* partly due to many such interventions being developed by commercial companies [10]. Fewer still have sought to include “end users” in usability testing.

The UK government’s “Five Year Forward View” encourages and enables individuals to take greater responsibility for their health through the use of eHealth or mobile health services [11], a sentiment that is mirrored in most high-income nations. This drive has become more pressing due to the impact of COVID-19, which has altered care models for people with diabetes (PWD) [12], necessarily shifting the focus towards remote care [13]. Among all chronic conditions, diabetes is possibly the most amenable to the use of DHIs in scalable follow-up care [14,15]. Many of the challenges faced by people in managing their diabetes occur “in the moments of everyday life” [15] when a DHI has the potential to deliver targeted and timely assistance.

Considering how crucial usability is for a DHI, it is concerning that a recent evaluation of four top-rated diabetes apps found that they all suffered from usability problems, many of which were “very serious” [16]. User-centered design of health interventions explicitly involves end users in their design, development, and evaluation [14,17]. This approach has the potential to produce more acceptable and usable DHIs [18] by ensuring from the outset that an intervention is targeted to end users’ needs [19,20]. The use of iterative design cycles, which include end users at each stage of product development, is also recognized as being important in developing usable DHIs [10]. Gaining a good understanding of the context in which specified users will interact with the product is also important [21].

Sittig and Singh’s sociotechnical model [22] was developed to allow the social context of digital health care tools to be linked to the technical component, and it recognizes that the two components influence one another [23]. It has previously been adapted by others to examine a range of different health care technologies, including patient-facing portals and health apps [24-26]. It allows for different sociotechnical dimensions to be dismantled for the purposes of examining them but also encourages consideration of the relationships between dimensions [23]. The sociotechnical approach encompasses a human factors engineering approach, which attempts to optimize users’ performance of tasks whilst making allowances for human capabilities and limitations in complex environments [27].

In recent times, it has become possible for artificial intelligence (AI), including machine learning (ML) algorithms, to underpin DHIs. ML algorithms analyze large data sets to detect patterns in data [28]. They can generate predictive models, the outputs of which can support decision-making by users [29] and include predictions of future risks. Effective communication of risk within a DHI must take into account end users’ health literacy and numeracy [30]. In addition, DHI interfaces should adhere to evidence-based recommendations for the presentation of complex risk information to patients (eg, use of plain language and use of absolute rather than relative risk) [26,30].

My Diabetes My Way (MDMW) [31,32] is a DHI (interactive website and app) for PWD and their carers. It contains resources, including tailored information based on users’ health/lifestyle data, interactive educational resources, and videos, as well as access to users’ clinical data. MDMW has been used in Scotland since 2008 and has more recently (since 2018) been deployed in several National Health Service (NHS) Trusts in England (ie, Somerset, Northeast London, Lancashire, South Cumbria, Cheshire, and Merseyside), where it is known as MyWay Diabetes. MDMW takes a subset of data from primary and (where possible) secondary care. These include key diabetes indicators (eg, glycated hemoglobin [HbA_1c_], blood pressure, and BMI), as well as eye and foot screening results, medication, and clinical correspondence. It provides users access to these records, as well as tailored advice and targeted resources based on each user’s status. History graphs permit individuals to interrogate their data and progress over time. One area of the site (the “managing your condition” page) alerts users to missed screening visits (based on the Diabetes UK “15 Healthcare Essentials” [11]). Patients can manually enter home-recorded data (eg, weight, blood pressure, and blood glucose) and set their own health and lifestyle goals.

An AI-augmented version of MDMW is being developed through linkage to the MyDiabetesIQ (MDIQ) analytics and reporting engine. MDIQ is linked to a knowledge base of approximately 70 validated machine learning models. It uses information from health care records (ie, linkages with hospital/general practitioner information technology systems) and home recordings (eg, Fitbit activity and home blood glucose data), driving predictive analytics. MDIQ models were developed in two ways: (a) using individual literature-published models and (b) meta-models derived from literature-published models, but delivering improved performance. Models were revalidated and tested in Scottish diabetes data sets, including NHS Greater Glasgow and Clyde (n=105,000) and Northwest London data sets (n=145,000). Proposed novel features underpinned by MDIQ include: (1) presenting users with their predicted risk of diabetes-related complications (based on their clinical and lifestyle data), (2) allowing users to visualize how lifestyle changes could impact their risk, (3) and providing ongoing, tailored feedback on progress toward users’ own goals.

This study aims to provide an overview of the iterative design process of the enhanced interface of this DHI and how human factors have informed system development using the sociotechnical model as a theoretical framework [22]. MDIQ AI models underpinning these additional features will be described in greater detail in a separate publication.

## Methods

### Focus Groups

Potential focus group participants were identified via local patient and public involvement (PPI) groups whose members had previously expressed an interest in taking part in diabetes-related research and who had consented to be contacted. Initial email contact was made by local research nurses. Interested patients were directed to contact the researcher for further information and to complete the consent process. Two of the authors (CS plus one other) moderated the focus group sessions, which were held in meeting rooms within a local private venue or hospital. Sessions were guided by an interview schedule (Multimedia Appendix 1) and screenshots of early designs (for a new-look homepage, goal-setting area, and novel risk prediction tool).

Focus groups were audio-recorded and later transcribed verbatim. NVivo 12 software (QSR International Ltd) was used to organize and code the textual data, which were analyzed using inductive thematic analysis [33]. In an effort to reduce bias, qualitative data were coded by 2 authors, a researcher (CS) and a clinician (NC). Any conflicts in coding decisions were reviewed by both and resolved by discussion and consensus [34].

Where participants are quoted in the text, they are referred to by the number of the focus group they attended and their participant number (eg, FG1P1 = focus group 1, participant 1), along with their sex, age, and diabetes type.

NHS Health Research Authority Research Ethics Committee approvals were obtained (IRAS number: 258231).

### Think Aloud Workshops

Focus group participants were invited back to attend workshops to user test a prototype site, the development of which was guided by focus group findings. Not all participants returned; therefore, several additional participants were recruited from the original PPI list. Workshops followed the “think aloud” method [35,36]. In this method, the participant is asked to explain their thinking, opinions, actions, and reactions as they perform several prescribed tasks [9]. Tasks given to users (Multimedia Appendix 2) represented common actions for which the system would eventually be used. Several tasks were similar to actions that participants would be familiar with when using the existing MDMW site. Other tasks were novel, relating to unique MDIQ-augmented features. All tasks were validated by 2 of the authors (CS and NC) and collaborator Louise McIver.

**Figure 1:**
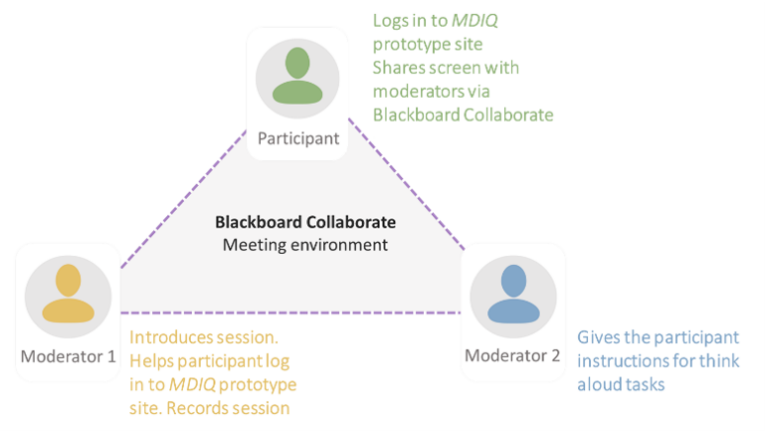
Schematic showing how think aloud method was performed remotely, using Blackboard Collaborate Ultra for the meeting environment. MDIQ: MyDiabetesIQ.

Think aloud sessions took place in August 2020 and were conducted remotely due to COVID-19 restrictions at the time of the sessions. A schematic of the set-up for remote user testing is shown in Figure 1. Each participant was sent a Blackboard Collaborate link several days prior to their session. Blackboard Collaborate was chosen because no special software download was required by participants, who could then easily screen share. Sessions were recorded by the moderators, with participants’ screens and speech captured simultaneously. Two moderators were present in each session: one (CS) introduced the activity, recorded the session, and made notes; the other (Louise McIver) gave instructions for think aloud tasks and encouraged the participant to continue to speak if they became quiet for too long.

Video recordings of sessions were later transcribed verbatim, and each transcript was annotated with observations (eg, position/activity of the user’s cursor). A combination of deductive and inductive thematic analysis was employed [33]. Text fragments relating to usability problems were mapped onto all relevant dimensions of the sociotechnical model [22] (ie, human-computer interface, clinical content, workflow and communication, people, and hardware and software), which became the major themes. Within each of the dimensions, an inductive approach was used to identify subthemes that were generated from the data. Qualitative data were coded by 2 authors, a researcher (CS) and a clinician (NC). Any conflicts in coding decisions were reviewed by both and resolved by discussion and consensus [34].

Where participants are quoted in the text, they are referred to by participant number (eg, TA_P1), along with their sex, age, and diabetes type.

The think aloud component of the study was deemed to be “service evaluation;” therefore, ethics approval was not required.

## Results

### Focus Groups

#### Focus Group Participant Demographics

Two focus groups were conducted in November 2019 and January 2020 in two different regions of Scotland, with a total of 8 participants. There were 2 participants in the first group and 6 in the second group, giving a total of 8 participants. Most participants were male (6/8, 75%), a majority (5/8, 63%) had type 1 diabetes (T1D), and 3 (37%) had type 2 diabetes (T2D). The mean age was 64 years (range 49-83 years), and the mean time since diagnosis was 22 years (range 5-58 years). The average recording length of focus group sessions was 64 min.

#### Thematic Analysis

**Figure 2:**
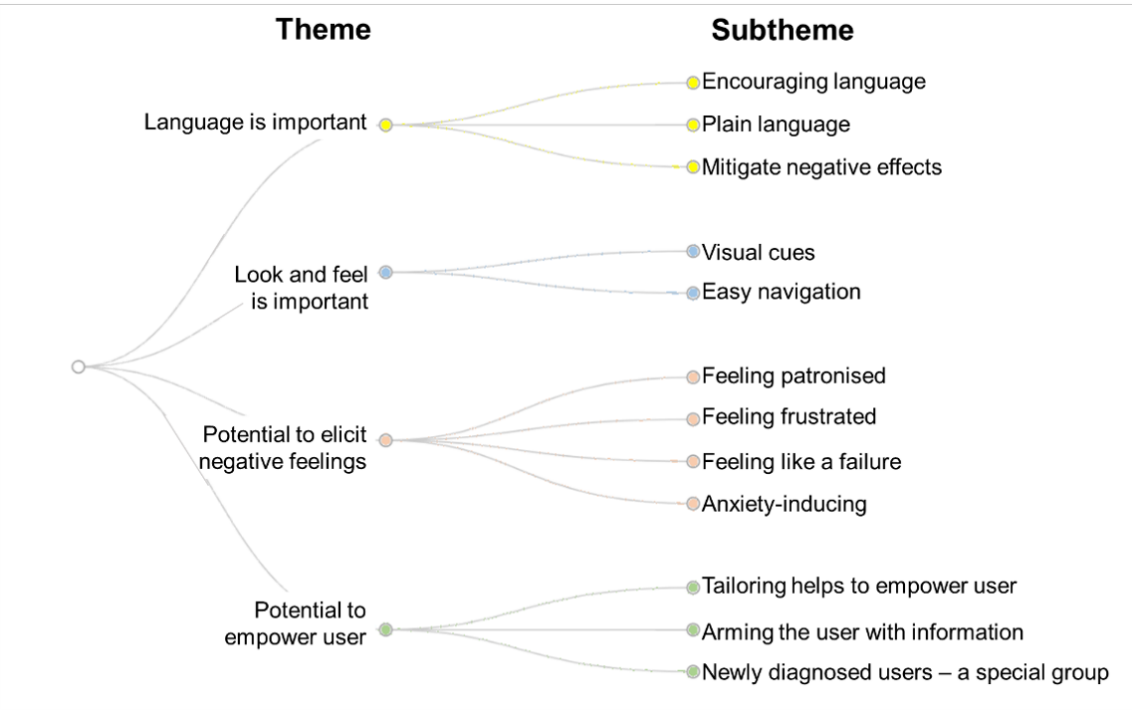
Themes and subthemes identified from focus group discussions.

Themes and subthemes generated from focus group transcripts through inductive thematic analysis are highlighted in Figure 2. These are discussed below in relation to novel features of the MDIQ-augmented site.

##### Potential to Empower Users

The majority of participants (5/8, 63%) felt that some proposed new MDMW features had the potential to empower users. For example, one novel feature described to participants during focus groups was the potential for users to set their own goals via the system and then receive ongoing tailored feedback on progress made toward goals. Two (25%) participants felt that this could help to empower users. An example of this sentiment is given below:

You’d set your weight goal and then say ‘every week I’m going to step on the scales,’ and then you’d put it in. It could be a useful tool as a specific thing. Especially if your doctor has wagged their finger at you.

FG2P1, male, age 58, T1D

In the case of a proposed new risk prediction tool, which would display a user’s future risk of developing diabetes-related complications and allow them to alter sliders representing lifestyle choices to see how this impacts their risk, 5 (63%) participants felt that this had the potential to empower users by arming them with information, allowing them to take control:

You’ve got the recipient taking decisions and setting their own targets...That might go down well with some folk. Very much so.

FG1P2, male, age 83, T2D

…that’s what you want. With diabetes, in particular, you want the patient to have control themselves.

FG1P2, male, age 83, T2D

Those participants who were positive about the risk prediction tool saw it as a way of facilitating users setting appropriate goals.

##### Potential to Elicit Negative Feelings

Despite the acknowledgment that users could be empowered by proposed features, 6 (75%) participants also felt that these could have undesirable effects such as eliciting negative feelings, including users becoming anxious or feeling as though they had “failed.” In the case of goal-setting with ongoing feedback, there were concerns about the consequences if a user did not achieve their goals and that this might produce feelings of failure:

I’m always wary about giving folk targets—what if they don’t make the target? There is a consequence.

FG1P2, male, age 83, T2D

I looked at it last night, and it said, ‘your height is this, and your weight is that.’ It says, ‘you are overweight.’ Now I was overweight by 200g! You know? But it said, ‘You are overweight. You’ve failed. You’ve failed.’ And that’s down to the language that’s in there. (referring to how the current MDMW system reports the user’s current weight).

FG2P1, male, age 58, T1D

It was pointed out by 3 (38%) participants that goals should be “achievable” and “safe” and that some guidance may be needed on what constitutes a realistic goal. This would help to mitigate against risks:

Yes, because they could be setting themselves up for failure just by making it [the goal] unrealistic.

FG2P3, female, age 62, T1D

For those of us who are patients—we should agree the goal with the clinician. My goal is: ‘I want to shave down my HbA_1c_ or lose a few pounds or drive down my cholesterol’ or whatever it might be. If they say, ‘yes, you could maybe do that in the next 4 months’ [sic]. I think it could be dangerous if you could randomly set your own goals.

FG2P1, male, age 58, T1D

Although recognized as potentially empowering for users, the risk prediction tool was seen by 4 (50%) participants to have the potential to cause anxiety (or to exacerbate existing health anxieties) due to users’ risks being revealed to them:

You can have a toggle to say ‘I don’t want to see that,’ but again if someone is worried—is a natural worrier—then they would click ‘yes, I want to see that kind of information’ and then it’s a positive—or a negative—feedback.

FG1P1, male, age 62, T2D

If folk are prone to worry about things, it could make it worse. But for most of us we’re quite pragmatic about it when you’ve had diabetes for a while.

FG2P5, female, age 49, T1D

Newly diagnosed users were regarded by 4 (50%) participants as a special group, who may feel more worried than other users when their future risks are revealed to them. However, it was also felt that they could be the group who could benefit the most from this information:

New folk—it might scare them a bit, but…you can’t have too much information.

FG2P5, female, age 49, T1D

For newly diagnosed people, it would be really useful, and I would have found this—yes scary—but still giving me more information.

FG2P1, male, age 58, T1D

##### Language Is Important

In terms of mitigating any anxiety arising from using the risk prediction tool, 5 (63%) participants agreed that the use of positive and encouraging language would be vital:

I think you have to say that because it is lifestyle parameters, all of those can be addressed.

FG2P1, male, age 58, T1D

Yes, so it’s not all doom and gloom. This is what it would look like if you carry on, but there is a process for improving on that.

FG2P2, male, age 66, T1D

In order to further mitigate against anxiety about complications risk, 3 (38%) participants felt that users should be provided with extra guidance, such as information or a link alongside the risk prediction tool, directing them to mental health advice or suggesting that they speak to their health care provider (HCP) if they are concerned about what they have seen:

People choose whether to see that or not, but if they do choose to see it, and the results are bad, then you could maybe put a link in or something to take them to the mental health questionnaire.

FG1P1, male, age 62, T2D

How difficult would it be [for the site developers] if you’re on it and you want to click on a link for support, like your diabetic nurse or your clinic?

FG2P6, male, age 64, T2D

##### “Look and Feel” of the Site

The “look and feel” of the site was also considered important by 4 (50%) of the participants. Discussions on this theme fell into two subthemes: (1) that images and icons would be preferable to large blocks of text (or lists of data) and (2) that navigation should be easy and intuitive:

…you end up with a string of menus. You look for your own measurements—there’s eight or nine of them, and you work your way down them, and ok by the time you get to number nine you’re beginning to forget what was number one! (referring to current MDMW site)

FG1P2, male, age 83, T2D

If you were going into the front page with your five circles, and you tapped into one of the reds, and you got your graph, that would make sense. (responding to screenshots of possible new designs)

FG1P2, male, age 83, T2D

It was also felt, by 2 (25%) participants, that “jargon” should be avoided as much as possible and that any medical terms that are included should be accompanied by additional explanations:

So I think alongside each complication, you could have an explanation of what that is…It should say what it specifically is—what it affects, I think it should give a bigger explanation.

FG2P2, male, age 66, T1D

It needs to assume a low level of jargon knowledge.

FG2P1, male, age 58, T1D

### Development of the Prototype Site

User perceptions and preferences collected during focus group discussions were considered and discussed by the researcher (CS), several diabetes clinicians (NC and DW), and the lead platform developer (DB). Those that were felt by all members of the team to be useful and feasible were incorporated into the prototype site. In this way, user insights directly influenced prototype site development. For example, participants’ desire for clear visual cues and easy navigation was addressed by giving the homepage a “clean” look, with colored blocks (containing representative icons) for each test result/lifestyle item. When clicked, the blocks took the user to further details about each item. In comparison to the current MDMW site that participants were used to, fewer clicks were required to reach frequently sought information (eg, HbA_1c_ history graphs and eye and foot screening results).

Participants’ desire for positive language was also heeded. An example in the risk prediction tool was the addition of a heading: “Let’s reduce your risk.” In response to participants’ demand for plain language (and for additional explanations where medical terms were necessary), diabetes-related complications in the risk prediction tool were condensed into a single (combined) risk of developing complications, with an information icon alongside to explain what kinds of complications this pertains to. The positive reaction to the risk prediction tool as a means of fostering suitable goals was harnessed by adding the option (via a button) to link outputs from this page to the goal-setting area.

### Think Aloud Sessions

#### Think Aloud Participant Demographics

Seven participants took part in think aloud sessions with the prototype site. Of these, most were female (4/7, 57%), and most had T1D (5/7, 71%). The mean age was 52 years (range 35-62 years), and the mean time since diagnosis was 23 years (range 4-37 years). Sessions had an average length of 41 minutes.

#### Thematic Analysis

**Figure 3:**
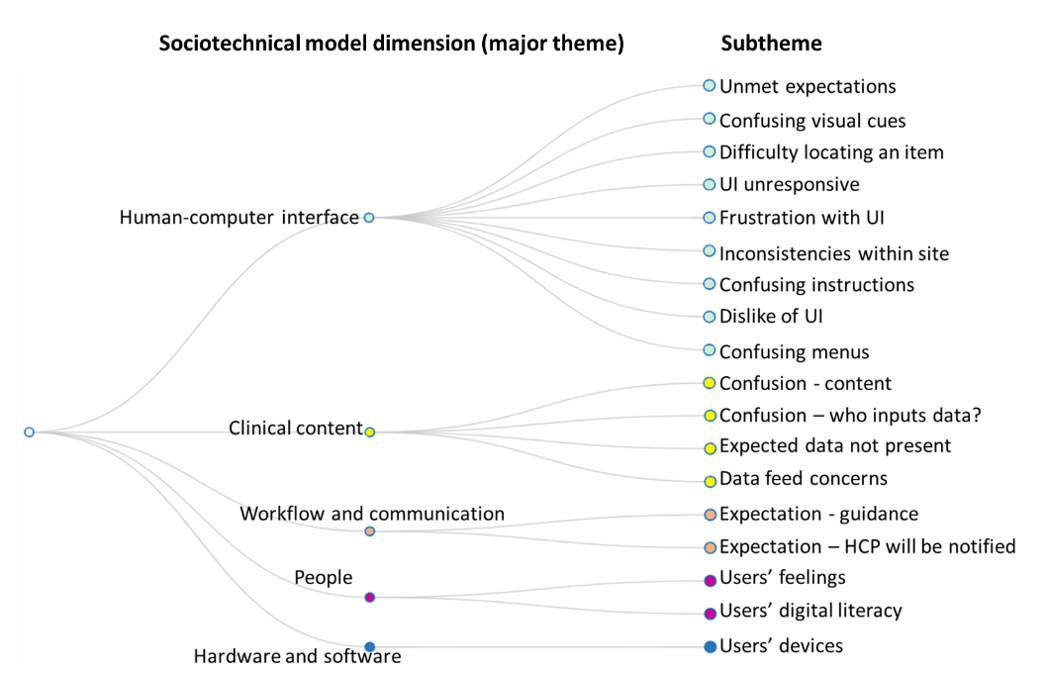
Thematic categories of think aloud transcript data. The major (deductive) themes were the five relevant dimensions of the sociotechnical model. Subthemes were (inductive) themes within each dimension that were generated from the data. HCP: health care provider; UI: user interface.

The 126 pertinent text fragments were mapped onto the 5 relevant sociotechnical model dimensions, which formed the basis of the major (deductive) themes of the analysis. Through an inductive approach, subthemes were generated from the data within each of the 5 dimensions. Some fragments were coded into more than one thematic category, resulting in 136 fragments included in the analysis. Categories from the thematic analysis are summarized in Figure 3.

Usability issues identified from think aloud user testing are summarized below and are discussed in relation to each of the sociotechnical model dimensions.

##### Human-Computer Interface

The majority (79/136, 58%) of text fragments from think aloud transcripts mapped onto the human-computer interface dimension of the sociotechnical model. Within this dimension, 9 subthemes were identified (Figure 3). One of the most prevalent of these, with 17 (13%) text fragments, was “confusing visual cues.” Examples include users not realizing that an item on a page was clickable and it not being obvious what the different areas of a page represent:

As I hover over it, it’s ‘coming up’ which suggests it is clickable, but it’s not that obvious. I wouldn’t…it’s not that obvious. (referring to an object’s hover state)

TA_P1, female, age 35, T1D

One of my first thoughts was: it's not obvious. It needs to say 'current' and then 'future.' (referring to left- and right-hand sides of the risk prediction tool page)

TA_P2, female, age 49, T1D

Another common theme, 15 (11%) text fragments, within the human-computer interface dimension was “unmet expectations.” An example (in the risk prediction tool) was the expectation by users to be able to select a value for lifestyle/clinical variables and type in a replacement value, rather than changing this using a slider:

It doesn’t seem to highlight ‘HbA_1c_’. Now with the ‘smoking’ you had the wee bubble so you could change it. And you had a slider bar for ‘activity’. But with ‘HbA_1c_, you’ve got nothing, so I don’t know whether you click on it.

TA_P4, female, age 49, T1D

“Inconsistencies within the site” was also a recurring theme within the human-computer interface dimension, 6 (4%) text fragments. An example was that some blocks on the homepage altered when hovered over (a shadow appeared around the edges), indicating to the user that they were clickable. Other blocks did not possess altered hover states, leading some users to assume these were not clickable.

Some items were unresponsive, leading to several users becoming frustrated. An example was a slider in which the value did not change as the user moved the slider button along and only refreshed once the button was released:

Right so when you’re actually clicking on the slider, although you can move it, it’s not moving with the number.

TA_P2, female, age 49, T1D

It doesn’t change as you move the slider. No…It’s very, very fiddly.

TA_P4, female, age 49, T1D

##### Clinical Content

Six text fragments (4%) referred to “confusion over who inputs data” (ie, whether it is the user or their health care team who should do this). Within the “managing your condition” page (a checklist of diabetes tests, checks, and services that all people with diabetes should receive), some items (eg, “have your legs and feet checked” and “have your blood pressure measured”) are automatically updated by the system once the user has attended a screening appointment, while others (eg, “receive high-quality care if admitted to hospital” and “get emotional and psychological support”) must be updated by the user.

I thought that someone at the clinic would be doing that for you…it would be quite nice to have something to say that can be completed by the patient.

TA_P5, female, age 51, T1D

Within “clinical content,” there was also a subtheme of “expected data not being present.” For example, 2 (29%) participants expected access to retinal scan images via the retinal status page.

##### Workflow and Communication

Three of the participants (43%) wrongly expected that their HCP would be notified when they entered data into the system. For example, within the “managing your condition” page, the user can tick “received” or “not received” alongside a checklist item such as “get emotional and psychological support.” Some users incorrectly assumed that ticking “not received” would alert their GP that they needed further support. This is not the case, as the checklist is intended to serve only as a reminder for the user:

So basically it gives the patient an opportunity to ask for something which they think could be useful to them.

TA_P3, male, age 62, T2D

##### People

Four participants (57%) pointed out that users with limited digital skills might find it challenging to interact with some aspects of the DHI. This was mentioned in relation to using the risk prediction tool (which involves the user interacting with several sliders and toggles and interpreting risk prediction outputs), as well as in relation to navigating from the homepage to other pages, as in the following example:

Having green boxes and red boxes is really good. I’m all for colors and highlighting things. Especially for people who are not as tech-savvy, as I am not.

TA_P4, female, age 49, T1D

Sentiments that the risk prediction tool could potentially cause anxiety, which had been expressed during focus groups, were reiterated by 4 (57%) think aloud participants:

It might put a lot of pressure on some people because it's not as easy to change your HbA_1c_ as just moving a slider.

TA_P5, female, age 51, T1D

It's important it’s not rolled out to some patients who have a high risk and who have little or no opportunity to address that.

TA_P6, male, age 58, T1D

Two participants (29%) commented that additional information that appears beneath page titles (eg, the HbA_1c_ page and weight and BMI page) explaining these medical/lifestyle terms were superfluous and could make users feel “patronized”:

The banner headline at the top. It's very patronizing. Stating the bleeding obvious, you know? For anyone who's been diagnosed for a while and has a bit of understanding about the condition.

TA_P6, male, age 58, T1D

##### Hardware and Software

Three text fragments (2%) referred to users’ experience of the site being dependent on the device they use. One example is a list of screening results that appeared at the bottom of the retinal status page, which one participant felt might be missed by someone accessing the site using a mobile phone:

Unless you know to scroll down you might miss that bit about retinopathy. I suppose if you were looking on a phone, as well, you might not see that.

TA_P5; female, age 51, T1D

### Development of the Final Site

User insights gained from focus groups and think aloud workshops with the prototype site were discussed among the researcher, clinicians, and developer (CS, NC, DW, and DB), and those that were feasible were used to inform the development of the final (live) MDIQ-augmented MDMW site. The resulting changes included the use of more positive and encouraging language throughout the site and mitigating users’ anxieties. An example of the latter is a line of text added to the risk prediction tool suggesting that the user talks to their health care team if they are concerned about anything they have seen (Table 1). A user’s risk of developing complications will be presented as a single “combined risk,” with a breakdown of what those complications are (for users who wish to find out more). A simple explanation of what probability means will also be explained in a text box via an information icon (Table 1). A video will be added alongside the risk prediction tool, explaining how to use the tool, defining the terms used within the tool, and what the outputs mean. This will assist users with low digital and health literacy and will satisfy the need for plain language. The risk prediction tool will be displayed only after a new user’s third visit to the MDMW site, allowing newly diagnosed users to become familiar with the site (and with having diabetes) before being presented with their complications risk forecast.

In the final site, if a user saves the lifestyle settings in the risk prediction tool as goals, these will be saved to the goal-setting area of the site. Within the goal-setting area, such goals will appear alongside links to resources on setting achievable goals, as well as links to targeted education resources (eg, resources on smoking cessation for those who set a goal to “give up smoking”).

Several examples of unclear visual cues have been addressed in the final site. Within the risk prediction tool, the representation of “current risk” on the left-hand side of the risk prediction tool and potential “future risk” on the right-hand side, which had been unclear to several think aloud participants, has been made explicit (left side has been made smaller, and there are now clear labels above each side). Signposting to sliders within the risk prediction tool has also been improved (Table 1). Sliders have been made more consistently styled and more responsive (changing in real time as the slider button is dragged) in comparison with those in the prototype site.

**Table 1:** Examples of how insights gained during focus groups and think aloud workshops informed the development of the final MDIQ^a^-augmented MDMW^b^ site.

Insight from focus groups/think aloud workshops	Resulting change in live MDIQ-augmented site
User being shown their risk of developing diabetes-related complications could cause anxiety.	Addition of text box to the risk prediction tool: 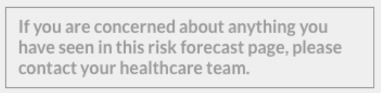
Plain language needed, explain terms used	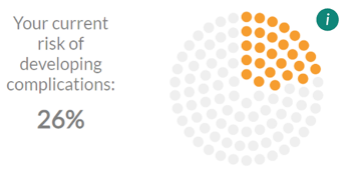 Information icon text, when clicked: 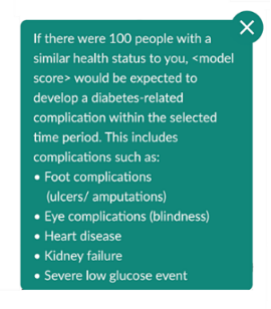
Clearer instructions needed	For example, addition of text directly above the sliders on the risk prediction tool: 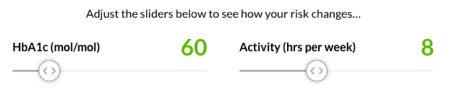

^a^MDIQ: MyDiabetesIQ.

^b^MDMW: MyDiabetes My Way.

## Discussion

### Principal Findings

This study has demonstrated that end-user involvement at each iterative stage of the design of a DHI can help to prioritize users’ requirements throughout the design process. This novel version of an existing DHI for diabetes (MDMW) will be underpinned by an ML engine (MDIQ). Before developing a prototype interface, developers were made aware of user perceptions and preferences collected during initial focus groups. The resulting prototype was then user tested through the think aloud method, leading to the development of a new live MDIQ-augmented MDMW site, soon to be released. This work paves the way for a future iteration of this user-centered design process, which will entail large-scale real-world testing of the live site.

The sociotechnical model used here as a framework for the thematic analysis of think aloud data [22] needed to be adapted, with 3 of the model dimensions (“internal organizational policies, procedures, culture and environment”; “external rules, regulations, and pressures”; “system measurement and monitoring”) not being relevant in the context of this patient-facing DHI. These dimensions are more applicable in clinician-facing health information technology used in formal health care settings, in which the model was originally conceived [22]. Think aloud data were therefore mapped onto the remaining 5 dimensions of the model (“human-computer interface,” “clinical content,” “workflow and communication,” “people,” and “hardware and software”), and subthemes were generated from the data.

It is useful to consider our findings in relation to each of the dimensions of the sociotechnical model individually. However, there is much interplay between dimensions. For example, many of the subthemes described within the human-computer interactions dimension (eg, “unclear visual cues” and “unclear instructions”) would be compounded for a user who has limited digital skills (people dimension). Confusion over who inputs data (clinical dimension) could be avoided by addressing issues around human-computer interactions, including visual cues and instructions. Understanding the connections between the model dimensions can help developers find solutions that don’t focus on one dimension while ignoring the impact of a given solution on others (ie, unintended consequences) [37]. This can additionally help developers to understand the wider sociotechnical structures already in place that could aid or constrain adoption of the technology under development [23] (eg, realizing that cues might be obvious to users who are used to interacting with online platforms but may not be obvious to less technically literate users). Awareness of these interactions between dimensions can help to make the site more usable for all users.

This study has demonstrated that viewing usability problems through a sociotechnical lens, and considering links between sociotechnical dimensions, can help foster the development of a more acceptable DHI. Findings from prototype usability testing are being used to inform the development of the final site, such as when an unclear visual cue was identified (human-computer interactions dimension), it was amended to address concerns regarding digital literacy (people dimension). Similarly, users expressed some confusion regarding which data items were updated automatically and which items were to be updated by the user (clinical dimension). There was also confusion over which self-entered data items were fed back to the health care team (workflow and communication dimension). The user interface will be amended to clarify both and to mitigate against clinical risk.

This study found that participants were more receptive to users setting goals based on the outputs generated by the risk prediction tool compared to straightforward goal setting, where a user simply sets a goal for themselves (“potential to empower users” theme). Some participants were concerned about users setting “unrealistic” goals, thereby “setting themselves up for failure,” or setting “unsafe” goals (people dimension). In response, the DHI now incorporates a link to the goal-setting area within the risk prediction tool and has been amended to highlight the need to set achievable goals, with the aim to provide a “scaffold” to foster healthy goals whilst aligning these goals with the user’s accepted level of risk of diabetes complications.

Participant desires may not always be actionable, particularly when they rely on third parties whose working practices cannot be dictated by the DHI developers; for example, validation and approval of all user-set goals by clinicians were deemed desirable by some participants but would require clinicians to have extra time and flexibility in their working practices to facilitate this (workflow and communication dimension).

The possibility of the system offering ongoing feedback on progress toward user-set goals was suggested to focus group participants. The platform will continue to be developed with the potential to incorporate goal-setting notifications and alerts via email and mobile devices in the future. Any such developments will need to address participants’ concerns regarding the potential to induce anxiety or feelings of failure in the event of a goal not being achieved.

During focus group discussions, most participants expressed a need for “plain language” (avoidance of medical jargon), as well as the addition of explanations alongside medical terms. This informed the decision to condense the initial information given to users, with additional information accessed via an information icon. There were, however, contrasting views around annotating items with further explanations, with some users feeling “patronized.” PWD are a diverse group, and preferences will differ amongst individuals. For example, the needs of someone with longstanding diabetes will likely differ from someone who is newly diagnosed (ie, participants identified this latter group as having a lot to gain from the platform). MDMW already encompasses some degree of tailoring (eg, diabetes type, cholesterol, and blood pressure). Further tailoring (eg, user preference, diabetes duration, etc) is technically possible, although there is the potential that in doing so, the platform may become overly complicated.

### Limitations of the Study

Attempts to recruit a more diverse group of participants via purposive sampling were not realized owing to poor response rates to the initial invite. The number of participants was relatively small and skewed towards older people with T1D who have had diabetes for many years. However, due to the rich data it delivers, the think aloud method requires only small numbers of participants, a suggested sample size of 5 to 8 participants, to uncover a high proportion of usability problems [9]. In addition, the participants were all considered “expert patients” whose experience provided valuable insights.

Social distancing necessitated by the COVID-19 pandemic resulted in the use of remote online user testing. This approach has known disadvantages, such as moderators needing to deal with unexpected technical issues during sessions and participants potentially facing a cognitively demanding environment (ie, navigating a video conferencing tool as well as testing the online intervention in question) [38]. Efforts were made to minimize these issues by giving clear instructions prior to sessions and by reassuring participants during sessions. In addition, remote online user testing provides several potential benefits: participants can be observed using the intervention in a more authentic context compared with sitting in a lab with researchers, and participants have more control over the session than they would in a lab setting (eg, muting their audio) [38].

### Conclusions

This study demonstrates that acting on the input of end users at each stage of development can help to build more acceptable DHIs, aligning features to users’ needs and avoiding unintended consequences that might cause disengagement. Good control of diabetes already relies heavily on self-management. The disruption of clinical services secondary to the COVID-19 pandemic only served to accentuate the importance of diabetes self-management. Digital tools such as MDMW (and particularly the MDIQ-enhanced version now being developed) can offer personalized support for self-management, alongside access to patients’ electronic health records in a user-friendly environment. These features can facilitate self-management, thereby reducing users’ risk of developing diabetes-related complications (with potential significant reductions in health care costs). This study serves as an exemplar of user-centered design that will ensure that MDMW is relevant and usable for people with diabetes.

## Appendix

Multimedia Appendix 1 Focus group schedule.

Multimedia Appendix 2 Tasks evaluated in the think aloud sessions.

## Abbreviations

AI: artificial intelligence
DHI: digital health interventions
GP: general practitioner
HbA_1c_: glycated hemoglobin
HCP: health care professional
MDIQ: MyDiabetesIQ
MDMW: My Diabetes My Way
ML: machine learning
NHS: National Health Service
PPI: patient and public involvement
PWD: people with diabetes
T1D: type 1 diabetes
T2D: type 2 diabetes
