# Supplementary material for: User-Centered Design of A Novel Risk Prediction Behavior Change Tool Augmented With an Artificial Intelligence Engine (MyDiabetesIQ): A Sociotechnical Systems Approach
Source: JMIR Hum Factors. 2022 Feb 8;9(1):e29973. doi: 10.2196/29973 (PMC8864521; doi:10.2196/29973)
Supplement: Multimedia Appendix 1 [file humanfactors_v9i1e29973_app1.docx]

### Appendix 1. Focus group schedule

1. How frequently do you use MDMW and what is the main reason you usually visit?
2. If you were to set a goal (e.g. to lose weight), would it appeal to have regular messages about your progress towards that goal (based on your data within the system)?
3. Would you want to be made aware of your risks of developing diabetes-related complications in the future? How would it make you feel to see your risks presented to you on the screen?
4. If there were a section of the site where you could visualise your lifestyle choices (e.g. weight/smoking status/HbA1c/cholesterol levels/activity levels) and see the impacts of making changes to these on your risk of complications in the future, would this help you with e.g. setting goals?
5. How should things be worded when talking to users about risk?
6. Can you identify any risks of using MDMW with the new features we’ve discussed? If so, how might these risks be mitigated?
7. Is there anything that would deter you from using the new features we’ve discussed?
