# Supplementary material for: User-Centered Design of A Novel Risk Prediction Behavior Change Tool Augmented With an Artificial Intelligence Engine (MyDiabetesIQ): A Sociotechnical Systems Approach
Source: JMIR Hum Factors. 2022 Feb 8;9(1):e29973. doi: 10.2196/29973 (PMC8864521; doi:10.2196/29973)
Supplement: Multimedia Appendix 2 [file humanfactors_v9i1e29973_app2.docx]

### Appendix 2. Tasks evaluated in the think aloud sessions

1. Locate and examine your HbA1c history graph
2. Find the page where you would add a weight measurement. Add a new weight measurement of 90kg
3. Locate the page where you can set a new goal for cholesterol. Add a goal of 3.5 mmol/L to be reached by [*future date*]
4. Locate the retinal status page. Check what your retinal status was on [*date in the past*]
5. Find the *‘Managing your condition’* page – explore and comment on this page
6. In the *Risk prediction tool*, find out what your risk of complications would be in 5 years, if your smoking status were to change from ‘smoking’ to ‘non-smoking’; your activity level were to change from ‘low’ to ‘medium’; and your HbA1c was brought down to 48 mmol/mol
